# Berberine as a promising natural compound for the treatment of periodontal disease: A focus on anti‐inflammatory properties

**DOI:** 10.1111/jcmm.17019

**Published:** 2021-10-30

**Authors:** Saeed Mohammadian Haftcheshmeh, Amir Abbas Momtazi‐Borojeni

**Affiliations:** ^1^ Department of Basic Medical Sciences Neyshabur University of Medical Sciences Neyshabur Iran; ^2^ Department of Medical Biotechnology, School of Medicine Alborz University of Medical Sciences Karaj Iran; ^3^ Iran's National Elites Foundation Tehran Iran

**Keywords:** berberine, inflammation, periodontitis

## Abstract

Accumulating evidence during the last two decades has addressed the potential anti‐inflammatory properties of berberine (BBR), a bioactive alkaloid compound isolated from *Coptidis rhizoma*, in controlling or treating several inflammatory diseases. Periodontitis is one of the most common chronic and serious inflammatory diseases, in which uncontrolled and unabated host immune responses against periodontopathic pathogens play critical and crucial roles in the disease pathogenesis. Hence, regulating inflammatory responses in periodontitis has a valuable approach and holds promise in treating periodontitis. For the first time, this paper reviews the evidence from in vitro and in vivo experimental models to explore the anti‐inflammatory effects of BBR in periodontitis and exhibits that BBR has the high potency to exert anti‐inflammatory effects by reducing expression and secretion of pro‐inflammatory mediators including TNF‐α, IL‐1β, IL‐17, RANKL, MMP‐2, MMP‐9 and MCP‐1. The BBR‐mediated anti‐inflammatory actions could translate into the inhibition of the periodontal tissues and alveolar bone destruction and the control of the disease in vivo. As the second aim of this paper, we also paid attention to the therapeutic potential of BBR in treating human diseases regarding its anti‐inflammatory properties.

## 1 INTRODUCTION

Periodontitis is the most common chronic inflammatory disorder of oral characterized by the uncontrolled inflammation in the periodontium and the tooth‐supporting tissues, which subsequently contributes to the destruction of periodontal tissues and alveolar bone.^1^ Meanwhile, in the severe form of the disease (aggressive periodontitis), periodontitis can affect systemic health by increasing the patients’ risk for several diseases such as rheumatoid arthritis (RA), atherosclerosis, cancer, adverse pregnancy outcomes and pneumonia.^1^, ^2^

In light of recent evidence in the pathogenesis of the periodontal disease, it is well‐documented that subgingival bacterial pathogens, *Porphyromonas gingivalis*, and other periodontitis‐associated species, by colonizing in the dental plaque elicit host pro‐inflammatory responses.^2^, ^3^ These pro‐inflammatory responses are complex and contain both protective and destructive responses.^4^ Although periodontal pro‐inflammatory responses are protective against periodontopathic bacteria, failure in the mechanisms that tightly regulate inflammation in the oral cavity results in the generation of destructive and unabated inflammatory reactions that mediate tissue damages.^4^ It is now well established that both innate and adaptive immune responses play crucial roles in the immunopathogenesis of periodontitis.^5^ Local inflammation is the main feature of periodontitis that is characterized by the infiltration of several inflammatory immune cells such as neutrophils, macrophages, dendritic cells (DCs), as well as B and T cells in the periodontium.^5^, ^6^ In innate immune responses, neutrophils and macrophages by producing different types of inflammatory cytokines such as tumour necrosis factor‐α (TNF‐α), interleukin 1β (IL‐1β), IL‐8 and monocyte chemoattractant protein 1 (MCP‐1) as well as by secreting several tissue‐degrading enzymes such as collagenases and matrix metalloproteinases (MMPs) play fundamental roles in the initiation of the periodontitis.^7^ In adaptive immune responses, inflammatory CD4^+^ T cells, especially IL‐17‐producing CD4^+^ T cells, are the main players in the development of the periodontium.^8^, ^9^ IL‐17 is one of the most important pro‐inflammatory cytokines that contribute to the destructive periodontal inflammation by recruiting neutrophils as well as by inducing the production of pro‐inflammatory mediators such as TNF‐α, IL‐1, IL‐6 and receptor activator of nuclear factor kappa‐Β (NF‐κB) ligand (RANKL), which eventually leads to the periodontal tissues destruction and alveolar bone resorption through osteoclast formation and activation.^10^

Understanding the mechanisms that contribute to the pathogenesis of periodontitis has led to the development of therapeutics aiming at treating or managing the disease. Therapeutic agents such as adjuvant administration of systemic antibiotics, corticosteroids and non‐steroidal anti‐inflammatory drugs (NSAIDs) are the fundamental property of periodontitis treatment; however, the main obstacle facing with long‐term use of these therapies is limitations and adverse effects such as microbial resistance, the influence on the entire microbiome of the human organism, infections, liver injury, gastrointestinal damage and heart failure.^11^, ^12^ Hence, there exists an urgent need to address alternative therapeutic agents, which may consist of synthetic or natural origin, for the treatment of periodontitis.

The past two decades have seen increasingly rapid advances in the field of pharmacology using naturally occurring medicine.^13^, ^14^, ^15^, ^16^ In this regard, mounting evidence has addressed the potential anti‐inflammatory properties of berberine (BBR), a bioactive alkaloid compound isolated from *Coptidis rhizoma*, in controlling or treating several inflammatory diseases. For the first, this paper provides an overview of BBR’s anti‐inflammatory effects and its therapeutic potential for the treatment of periodontitis.^17^

## 2 BBR: A POTENTIAL NATURAL OCCURRING COMPOUND

Berberine (benzyltetrahydroxyquinoline) (BBR) is an isoquinoline alkaloid originally isolated from the Chinese herb huanglian (*Coptidis rhizoma*).^18^ Studies over the past two decades have provided important evidence on the polytrophic pharmacological effects of BBR including anti‐microbial, anti‐oxidant, anti‐tumour, and anti‐inflammatory, and anti‐diabetic activities.^14^, ^19^, ^20^, ^21^ Interestingly, a considerable amount of research studies have been published on the immunomodulatory effects of BBR in the immune system.^19^, ^22^ These studies have confirmed that BBR’s anti‐inflammatory effect arises from its interaction with different types of immune cells including keratinocytes, epithelial cells, DCs, macrophages, mast cells, and T and B cells.^14^, ^19^ Interestingly, recent findings have been indicated that BBR as a pleiotropic molecule by interacting on several signaling molecules and transcription factors such as NF‐κB, activated protein 1 (AP1), janus kinases/signal transducer and activator of transcriptions (JAKs/STATs), mitogen‐activated protein kinases (MAPKs), and AMP‐activated protein kinase (AMPK) can effectively mediate its anti‐inflammatory roles in the immune system.^23^, ^24^, ^25^, ^26^, ^27^, ^28^

## 3 ANTI‐INFLAMMATORY EFFECTS OF BBR IN VITRO AND IN VIVO

**FIGURE 1:**
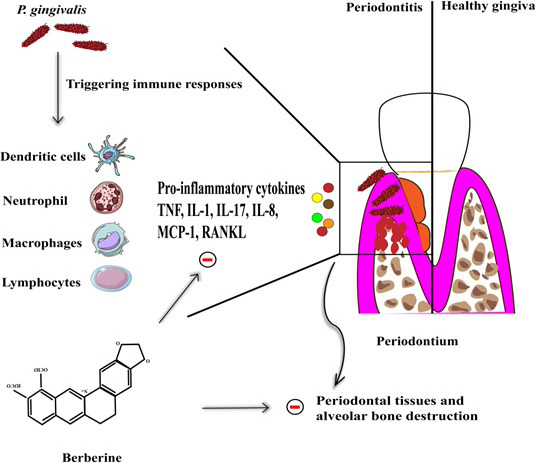
A schematic view of berberine's (BBR) anti‐inflammatory effects in periodontitis. BBR is capable of inhibiting the production of pro‐inflammatory mediators including TNF‐α, IL‐1β, IL‐17, MCP‐1 IL‐8, and RANKL as well as the expression and activities of MMP‐2 and MMP‐9 by inflammatory immune cells in the context of the periodontium

In the past two decades, a number of research studies have been carried out to investigate the anti‐inflammatory effects of BBR in periodontitis in vitro and in vivo. The potential anti‐inflammatory effects of BBR have been well indicated in an in vitro study, in which treatment with BBR dose‐dependently inhibited the activities of MMP‐2 and MMP‐9 in *P. gingivalis* lipopolysaccharide (P.g. LPS)‐stimulated gingival fibroblast cells and U937 macrophages.^29^ Moreover, in rats with periodontitis, BBR treatment attenuated periodontal tissue destruction by hampering the activation of MMP‐2 and MMP‐9.^29^ Another in vitro study also investigated the inhibitory effects of BBR on the production of monocyte chemoattractant protein‐1 (MCP‐1) and has found that the secretion of MCP‐1 from LPS‐stimulated human periodontal ligament cells (PDLC) significantly inhibited by BBR treatment.^30^ Importantly, MCP‐1 attracts neutrophils and other leucocytes to the site of inflammation, thus, BBR by inhibiting production of this key chemokine could suppress infiltration of leucocytes into periodontium.^4^, ^30^ To better understand the anti‐inflammatory effects of BBR in periodontitis, Jia and colleagues indicated that oral treatment with BBR (120 mg/kg/day) for 7 weeks markedly ameliorates alveolar bone resorption in a rat model of periodontitis.^31^ From the immunological point of view, BBR by attenuating the production of TNF‐α and IL‐17, as well as the number of IL‐17A^+^ cells in the alveolar bone effectively inhibited the local and systemic inflammation in the periodontitis rat model.^31^ TNF‐α is one of the most important pro‐inflammatory cytokines that are produced by macrophages and T helper 1 (T_H_1) cells and plays crucial roles in the pathogenesis of periodontitis. TNF‐α by inducing production of other pro‐inflammatory cytokines (IL‐1β and IL‐6) as well as by increasing vascular permeability and the expression of adhesion molecules recruits polymorphonuclear (PMN) cells into the tooth‐supporting tissues, where they release lysosomal enzymes, which contribute to the tissue degradation.^4^, ^6^ Notably, IL‐17 strongly induces the production of TNFα, IL‐1β, and IL‐6, mediating tissue and bone destruction via recruitment of neutrophils and also activation of osteoclasts.^4^ Another in vivo study by Gu and co‐workers showed that BBR has high potency to exert protective actions on alveolar bone loss related to inflammation in rats with ligature‐induced periodontitis.^32^ BBR treatment remarkably suppressed the infiltration of inflammatory cells in alveolar bone and attenuated the levels of pro‐inflammatory cytokines TNF‐α and IL‐1β.^32^ In this regard, the molecular mechanisms by which BBR exerts its anti‐inflammatory effects in periodontitis have been described in detail.^32^ BBR by increasing the protein expression of G protein‐coupled estrogen receptor (GPR30) effectively inhibited the activation of two important inflammatory signaling pathways involved in the pathogenesis of periodontitis including NF‐κB and MAPKs cascades.^32^ Further investigation also confirmed the anti‐inflammatory effects of BBR in periodontitis, as it has been shown that following treatment with BBR, the expression levels of pro‐inflammatory cytokines including TNF‐α, IL‐1β, and RANKL markedly downregulated in rat with periodontitis.^33^ In this study, proprotein convertase subtilisin/kexin type 9 (PCSK9), a key enzyme for the promotion of inflammatory responses, has been identified as a new target for BBR’s anti‐inflammatory action. In this regard, BBR treatment by reducing the production of PCSK9, which is subsequently followed by inhibition of inflammatory responses, remarkably alleviated P.g.‐induced periodontitis.^33^ As an important pathologic cytokine in periodontitis, RANKL, which is produced by activated T cells, mediates differentiation and activation of osteoclasts, leading to the alveolar bone resorption.^34^ Hence, BBR is capable of inhibiting bone destruction in periodontitis through hampering production of RANKL.^33^ Likewise, Yu et al hold the view that BBR exerts anti‐inflammatory effects in periodontitis, as they clearly indicated that BBR is efficacious to downregulate the expression of TNF‐α and IL‐β in periodontal tissues of rat with periodontitis.^35^ Table 1 provides an overview of the anti‐inflammatory effects of BBR in several preclinical (in vitro and in vivo) studies. Figure 1 also presents the BBR’s anti‐inflammatory effects in periodontitis.

**TABLE 1:** A brief overview of the anti‐inflammatory effects of berberine in vitro and in vivo

Type of study	Cell/animal model	Biological effects	Ref.
In vitro In vivo	Gingival fibroblast cells U937 macrophages Rat model of periodontitis	Inhibit the activities of pro‐MMP‐2, MMP‐2 and MMP‐9	[29]
In vitro	PDLCs	Reduce the secretion of MCP‐1	[30]
In vivo	Rat model of periodontitis	Attenuate the production of TNF‐α and IL‐17, as well as the number of IL‐17A^+^ cells	[31]
In vivo	Rat model of periodontitis	Reduce the levels of pro‐inflammatory cytokines TNF‐α and IL‐1β	[32]
In vivo	Rat model of periodontitis	Reduce the production of TNF‐α, IL‐1β and RANKL	[33]
In vivo	Rat model of periodontitis	Reduce the production of TNF‐α and IL‐1β	[35]

Considering aforementioned findings, it can be suggested that BBR by acting on inflammatory signaling pathways, which play key roles in initiating and promoting inflammatory responses in periodontitis, is capable of inhibiting inflammation that is represented by reduced production of pro‐inflammatory mediators and several tissue degrading enzymes.

## 4 ANTI‐INFLAMMATORY EFFECTS OF BBR IN CLINICAL TRIAL

What we know about the anti‐inflammatory effects of BBR is largely based upon the findings that emerged from in vitro and in vivo experimental studies. Besides, some clinical studies have also investigated the therapeutic potential of BBR regarding its anti‐inflammatory properties. In a randomized double‐blind phase I trial on patients with ulcer colitis, it has been shown that treatment with BBR (900 mg/day) for 3 months significantly decreased tissue inflammation. However, no significant changes in the tissue expression of NF‐κB and cyclooxygenase‐2 (COX‐2) were found following BBR treatment. Moreover, BBR had no significant effect on the plasma levels of pro and anti‐inflammatory cytokines including TNF‐α, IL‐2, IL‐6, IL‐8, IL‐4 and IL‐10.^36^ In another clinical trial, which set out to investigate the anti‐inflammatory effects of BBR in children with diarrhea, Chen et al found that oral treatment with BBR hydrochloride (0.2 gr/day) for one week significantly decreased the serum levels of pro‐inflammatory factors including TNF‐α and IL‐6.^37^

## 5 CONCLUDING REMARK

For the first time, the present study adds to the existing research investigating the anti‐inflammatory effects of BBR in periodontitis. Increasing evidence witnessed by the in vitro and in vivo experimental model clearly reveals that BBR as a pleiotropic agent is efficacious to exert anti‐inflammatory effects, as represented by the inhibited expression and production of several pro‐inflammatory mediators including TNF‐α, IL‐1β, RANKL, MCP‐1, MMP‐2 and MMP‐9, therefore, is capable of preventing the destruction of periodontal tissues and alveolar bone. The anti‐inflammatory effects of BBR might be mediated by its modulatory action on several inflammatory signaling pathways such as NF‐κB and MAPKs. Regarding the precise evidence emerged in clinical studies, it seems that BBR has therapeutic potential in treating several inflammatory diseases. Hereupon, it would be interesting to assess the anti‐inflammatory effects and therapeutic potential of BBR in patients with periodontitis.

## CONFLICT OF INTEREST

We wish to confirm that there are no known conflicts of interest associated with this publication and there has been no significant financial support for this work that could have influenced its outcome.

## AUTHOR CONTRIBUTIONS

**Saeed Mohammadian Haftcheshmeh:** Data curation (lead); Writing‐original draft (lead). **Amir Abaas Momtazi‐Borojeni:** Conceptualization (lead); Validation (lead).

## Data Availability

Data sharing is not applicable to this article as no new data were created or analysed in this study.
